# Using the participatory education and research into lived experience (PEARLE) methodology to localize content and target specific populations

**DOI:** 10.3389/fdgth.2022.992519

**Published:** 2022-10-21

**Authors:** Ian David Aronson, Alex S. Bennett, Mary-Andrée Ardouin-Guerrier, German J. Rivera-Castellar, Brent E. Gibson, Brittney Vargas-Estrella

**Affiliations:** ^1^ Technology-Based Education for Community Health (TECH) Lab, Department of Social and Behavioral Sciences, School of Global Public Health, New York University, New York, NY, United States; ^2^ OnPoint NYC, New York, NY, United States

**Keywords:** technology, intervention, community, substance use, opioids, injection, COVID-19, methodology

## Abstract

Technology-based behavioral health interventions offer potentially limitless opportunities to localize content and target specific populations. However, this ability to customize requires developers to make a wide range of decisions not only about who should appear on screen, but how each message should be refined to most effectively reach a particular group of intervention recipients. These issues become especially salient as interventions are scaled for delivery to multiple populations in different geographical locations or settings (e.g., a hospital emergency department versus the drop-in center of a community-based clinic), and in more than one language. To facilitate evidence-based development of customized, targeted intervention content, our team created a multi-step methodology over a series of NIH-funded research projects. The resulting Participatory Education and Research into Lived Experience (PEARLE) Methodology entails formative qualitative interviews to examine why members of a given population do not enact a specific health behavior such as HIV/HCV testing or vaccinating against COVID-19 (this step includes identifying potential gaps in related health literacy), followed by iterative evaluations of draft content designed to address these barriers, and extensive discussions with a Community Advisory Board. The final step is a clinical trial. PEARLE is designed to be highly flexible, adaptable to a variety of behavioral outcomes in clinical and community settings, and to create content in more than one language depending on the needs or preferences of a population. The current paper discusses how our team employed PEARLE to develop content in English and Spanish for our latest project, which is intended to increase COVID-19 vaccination uptake among people who inject drugs.

## Introduction

Technology-based behavioral health interventions offer valuable opportunities to help underserved groups in clinical and community settings. For example, adolescents and young adults frequently do not report risk behaviors because they fear being stigmatized by clinical staff, but a sample of New York City emergency department patients aged 13–24 provided detailed responses to very thorough substance use and sexual risk screenings *via* tablet computer because they did not fear judgement from a computer (1). For similar reasons, our team's research has shown participants may be more likely to accept HIV/HCV testing offered by computer compared to HIV or HCV tests offered face-to-face (2). Additionally, because technology-based intervention content can be carefully optimized, well-developed programs can ensure that content is delivered in high-fidelity to intervention design, unlike face-to-face interventions that can potentially prove more or less effective based on the training, experience, or skill of the person delivering them (3).

Perhaps most importantly, well-developed applications can localize content to increase acceptability and intervention effectiveness among those most in-need. Iterative development and optimization processes can potentially include, but are not limited to, adjustments for demographic differences (primary spoken language, race/ethnicity) and behavioral characteristics (e.g., creating one set of overdose prevention materials for people who inject heroin and another for people who sniff; or one video on the importance of HIV testing for men who have sex exclusively with male partners and another for men who have sex with both male and female partners). Developers may also choose to adjust for setting (i.e., content intended for patients in an emergency department might be very different than content intended for people seeking services at a community-based syringe service program). Content can also be adapted for different countries or geographical regions (people onscreen may speak with identifiable accents or use colloquialisms associated with specific places).

While this possibly infinite range of development options can lead to unquestionably localized content, it can also potentially overwhelm technology developers with the complexity of questions they need to answer. At the very least, technology-based intervention creators must decide who should appear onscreen, what these people should say, and in what type of setting they should appear. Additional related questions include whether people onscreen should be demographically concordant with intervention recipients (and if so, whether this concordance should be based on race and gender or on other characteristics), and whether featuring experts or community members onscreen will prove more effective. As noted above, the seemingly endless permutations can lead developers to spend exorbitant amounts of money (not to mention time) creating content for a project only to find their end-product does not align with the expectations of intervention recipients.

To address these issues, our team has developed a methodology over the course of multiple NIH-funded studies with vulnerable populations in the United States. The Participatory Education and Research into Lived Experience (PEARLE) methodology is designed to create highly engaging content by iteratively developing and refining materials in partnership with community members. In the current paper, we describe PEARLE in the context of our latest project, a technology-based intervention designed to increase vaccination against COVID-19 among people who inject drugs. As detailed in the following pages, our approach is intended to help developers localize content for any given population and can be adapted to encourage health behavior change far beyond vaccination.

## Foundations of the methodology

PEARLE builds upon two widely accepted models of health behavior: Bandura's Social Cognitive Theory (SCT), and Fisher and Fisher's Information, Motivation, Behavioral Skills model (IMB). Both offer important guidelines for intervention designs, and the selection of onscreen models. SCT (4) notes that before people change an aspect of their behavior, they must first decide that specific behavior is worth attending to. One way to encourage this attention is by presenting educational models that resemble viewers in terms of their age, sex, and status, as well as the types of problems and situations they face (5). IMB posits content should target specific populations and risk behaviors (6) and be made relevant to viewers' social settings (7). While accepting these recommendations as foundational, the PEARLE methodology entails multiple levels of community involvement to explore further questions that arise when SCT and IMB are operationalized. In short, the goal of PEARLE is to help intervention developers better understand how to create content that targeted recipients not only take notice of but fully attend to, in order to motivate measurable behavior change.

The first steps of PEARLE entail conducting qualitative interviews to: (a) identify why members of a specific population do not enact a particular health behavior; and (b) examine how these barriers can be addressed *via* custom authored technology-based interventions. The next steps involve iteratively developing and refining intervention content in response to new sets of interviews. Each of these first steps are conducted while regularly meeting with a Community Advisory Board (CAB) to discuss interview questions, then draft intervention content and findings. The final step entails a clinical trial to thoroughly examine the effects of intervention materials on measurable health outcomes. In prior projects, our team has used PEARLE to increase HIV testing among young emergency department patients (1, 8–11); to increase HIV/HCV testing and overdose prevention at a syringe service program outreach site (2); and to encourage people who inject drugs to more frequently carry naloxone in order to reverse overdose events (12, 13). As noted above, we most recently employed PEARLE in our project to develop content for an intervention designed to increase vaccination against COVID-19 among people who inject drugs who decline vaccinations when offered. So far, our work has focused on health behaviors with distinct and immediate behavioral outcomes [e.g., did the participant test for HIV post-intervention (1), did the participant accept a take home naloxone kit (14, 15), why do people decline to carry naloxone (13) or to accept a vaccination against COVID-19 (16)], but PEARLE can be readily adapted to other health behaviors and/or disease areas by following the steps below to develop and evaluate additional types of intervention content.

## Qualitative research

The start of a PEARLE project entails multiple phases of qualitative research: a *formative interview* phase with members of the target population to establish barriers to and facilitators of enacting the designated health behavior (in this case vaccination against COVID-19); a *formative evaluation* in which draft intervention materials are shown to a new set of participants to determine acceptability and potential effectiveness; and a *summative evaluation* in which actual intervention components are presented to an additional new sample of participants to examine which elements work best and which, if any, require further revision before implementation. This process has been adapted from the field of instructional design (17) in which content development is conceptualized less like a straight line, and instead is viewed as more of a spiral in which individual steps can be repeated as needed to evaluate and refine specific components before they are presented as finished products.

### Formative interviews, 1st wave of qualitative research

During our most recent implementation of PEARLE our team first interviewed 17 adults who had not vaccinated against COVID-19, and who reported injection drug use within the past 90 days. Participants were recruited at a syringe service program in New York City. Interviews were conducted by trained staff members in English and Spanish, depending on the preference of each participant. All study participants provided verbal consent prior to the start of the interviews. No identifiable data were recorded as part of the interviews.

Primary barriers to vaccination uptake included: fears of unintended/unknown side effects (including the false belief that a dose of the vaccine could infect people with COVID); mistrust of government and care providers; and potential dangers of the vaccine specific to people who have weakened health due to illicit drug use. Primary facilitators of vaccination included: protecting family and members of the interviewee's social networks; responding to the increased dangers of drug use during the pandemic; and messages that employ positive, reassuring tones to increase vaccination. Full details of this first set of interviews have been published previously (16).

### Formative evaluation, 2nd wave of qualitative research

Following the first round of interviews, our team began developing sets of storyboards (paper representations of intervention content) based on our findings. A key element of PEARLE is creating storyboards that can be developed quickly, at far less expense than a video or other intervention technology, and presenting these draft component designs to members of a target population to solicit feedback. While our preliminary interviews offered clear examples of barriers and facilitators, questions remained as to how we could most effectively depict these onscreen. For example, how could we most effectively portray the importance of vaccination as a way to protect a person's loved ones; or emphasize the supportive nature of staff at our partnering community-based organization?

To address fears of vaccination and mistrust of care providers, we created multiple storyboard panels depicting injections being administered. We also created panels featuring older people, racially and ethnically matched to our sample, who could potentially represent elderly family members who would be protected from the virus if participants vaccinated. Additionally, we included summaries of research that had recently been published showing unvaccinated people are more than twice as likely to be reinfected compared to those who have vaccinated, and people who are vaccinated are at least 10 times less likely to get sick or die from COVID-19 compared to people who are not vaccinated. Finally, we created a storyboard with an image of an older White man in a lab coat saying the flu kills roughly 30,000 people each year in the United States, but COVID killed more than 700,000 people in the US, and more than 4.5 million worldwide between January 2020 and October 2021, when the storyboard evaluations were conducted.

As part of our exploration of who should appear onscreen and what type of messaging they should deliver, we presented a picture of a young Black woman, wearing medical scrubs and a stethoscope around her neck, accompanied by text explaining that COVID-19 is caused by a virus called SARS-CoV-2, much like AIDS is an illness caused by a virus named HIV. Lastly, we paired an image of a Black man who appears to be between 50 and 60 years of age saying he had been hesitant to vaccinate because he feared side effects related to drug use, but that he did not actually feel sick after he vaccinated.

We then recruited a new sample of 20 participants who had not vaccinated against COVID-19 and who reported injection drug use within the past 90 days. We asked participants to review our storyboards, and then to participate in individual semi-structured interviews about their impressions of the characters and content, and whether or not they had additional suggestions for messaging deigned to encourage COVID vaccination.

All interviews were digitally recorded and then transcribed for analysis. Three team members, all trained in qualitative research techniques, used MAXQDA software for coding and analysis. Transcripts were analyzed by thematic analysis. Following completion of each audio-recorded interview, audio files were promptly transcribed by an outside service, REV.com. Our team met weekly to conduct analysis of the interview transcripts to identify broad thematic categories addressed in each interview about barriers and facilitators to vaccination. Thematic categories consisted of both *a priori* constructs (based on the aims of the study, a literature review, and the interview guide) and emerging themes (that were related to vaccine hesitancy, but not specifically anticipated). The major themes from the first set of interviews became initial thematic codes for the second and third sets of interviews. After each wave of interviews, additional codes were added during discussions with our community advisory board (described further below). Across waves, codes were compared for thematic consistency and discrepancies were processed during regular team meetings.

After each wave of interviews, a small subset was jointly analyzed by three team members, then codes and coding strategies were discussed by the larger team. Three team members, including two who also conducted interviews, analyzed the transcripts to illuminate some of the barriers to and facilitators of COVID-19 vaccination. We selected various quotes to illustrate dominant themes across interviews in the following pages.

Participants reacted strongly and negatively to the depictions of injection, although the images were intended to be reassuring. In previous interventions to increase HIV testing, participants told us that seeing video of rapid oral HIV tests being administered made the process less frightening and encouraged them to test (10). However, participants in the current study reported that to many people in our target population, images of a syringe may be perceived in a negative way that could create further resistance to COVID vaccination, as illustrated by the following quote:

We already know we're addicts, so why show the needle? Oh, I hate needles … behind closed doors, you're putting a needle in your arm, back, back, back, back, back, back, but don't tell anybody. Right? Don't show the needle. People don't want to see that.—Interviewee 30, Hispanic male.

Another participant cautioned us against mentioning AIDS in our materials because it is similarly charged with negative emotion and could potentially distract people from our intended messaging, or that people could misinterpret our materials as not applicable to anyone who is not HIV positive:

This one right here is very alarming too. They used the word AIDS, people who are, they hear that word, forget it, ‘cause it's, it's, um, it's, it's just pinpointing people with AIDS, it's not pinpointing generality.—Interviewee 26, Hispanic White male.

Other participants emphasized the importance of showing community members who talk about their drug use on camera. Including these community members would be especially valuable if they also discussed why they ultimately decided to vaccinate, despite initially fearing unintended vaccine side effects. A clear theme that emerged from our formative evaluation interviews was, as posited by both SCT and IMB, that watching people onscreen describe their experience with the desired behavior of vaccination could encourage intervention recipients to reconsider their decisions not to vaccinate, especially when the models emphasized the positive benefits.

“So, I was kind of scared to take it too, because I didn't know if I was gonna sick or anything. But now that I see that he took it, and it encouraged me more to want to get it. So, I won't have to get sick.”—Interviewee 31, Hispanic female. This would be particularly effective, participants explained, if we showed people onscreen who were recognizably part of their community. This was not necessarily defined by race or ethnicity, interview data show, but by how people onscreen dressed and how they spoke.

## Health literacy

Similar to our formative interviews, our storyboard evaluations indicate participants were ambivalent about vaccinating against COVID at least in part because they had heard so much conflicting information from so many different sources:

It came onto the media and then people said, “Well, what is this all about?” Because some say it's curable and some say it's not, and you know, some say the vaccine is working, some say it's not. So that's gonna discourage people from getting vaccinated.—Interviewee 21, Hispanic male.

Nonetheless, additional interview data from our sample of unvaccinated individuals show that learning specific details about the dangers of COVID from our storyboards encouraged some participants to consider vaccinating. Many reported not previously understanding the difference between COVID and influenza, “I didn't know that COVID and flu are not the same. I actually thought they were … Wow. That's crazy,”—Interviewee 22, Hispanic male. Others reported not knowing that COVID was much more deadly compared to influenza, and also more persistent:

That it killed 4.5 million people. 700,000 people in the United States. … That you get reinfected from it again. … I didn't know that. Like, I thought that once you had, you had, that was it.—Interviewee 32, Hispanic female.

### Community advisory board

The current project builds upon longstanding relationships with a community-based organization that provides low threshold services to people who use drugs. One key component of our collaboration includes monthly meetings with a CAB that we empaneled as soon as the project started. The CAB consists of four staff members from our partner community-based organization, several of whom also provided substantial feedback on the initial project protocol. The CAB also includes six participants who currently receive services from the organization. As our team noted in a previous publication (18), one of our chief goals is to create interventions in collaboration with participants and staff that can be administered with minimal disruptions to established workflows, and that are acceptable both to people who receive services and the staff who provide them. Put simply, we wanted to create a working environment free of judgement where collaborating partners can openly express their views while listening to those of others. All members of our CAB were unvaccinated against COVID-19 at the time the board was empaneled, so they were able to provide important insights on why members of our target population decline vaccination when offered.

These CAB meetings have continued through all phases of our project. In our first meetings we collaboratively revised study instruments (qualitative interview guides, demographic questions, substance use screenings, pre-post health literacy items) to ensure members of our target population would find them easy to understand and non-objectionable. We spent considerable time refining the substance use screening to reflect the depth and breadth of our population's knowledge (e.g., participants pointed out that Valium was incorrectly listed as a “sedative” in a screening created by the World Health Organization, and suggested it should actually be categorized as a benzodiazepine).

During initial CAB discussions our team emphasized that we wanted to hear member's candid perspectives on vaccination, that all perspectives are valid, and that we would not criticize their beliefs, even if they conflicted with mainstream or institutional views. We explained that a major goal of our PEARLE-informed study was to understand why people did not vaccinate, so that we could develop stronger intervention materials. Two main themes emerged during meetings that aligned strongly with findings from our preliminary interviews—CAB members feared unwanted side effects of vaccination, and were also wary of potential dangers that might be specific to people who use illicit drugs (e.g., drug use may have weakened their overall physical condition, making them more susceptible to untoward vaccination side effects). Discussions in our CAB meeting also revealed an incorrect yet widespread belief that vaccines were made from a live culture of the COVID virus, which could potentially lead to COVID infections among those who vaccinate. As a result, we chose to specifically address these items in our intervention video.

During the 4 months of CAB meetings that took place during the first two waves of qualitative interviews, we noticed that our full CAB (all of whom were unvaccinated at the time they joined) had received vaccinations against COVID-19. When we asked what contributed to their decisions to ultimately vaccinate, CAB members attributed the change to a combination of factors including: financial incentives (at the time, New York City was offering $100 to anyone who received a first dose); tightening restrictions that severely limited work, travel, and restaurant dining for unvaccinated people; and information they had learned through discussions at our CAB meetings. As we wrote in a previous publication, providing a safe environment for CAB members to express themselves without judgement, and encouraging them to voice their honest concerns about vaccination, may have encouraged people to reconsider their earlier opposition to vaccination (16).

**Figure 1:**
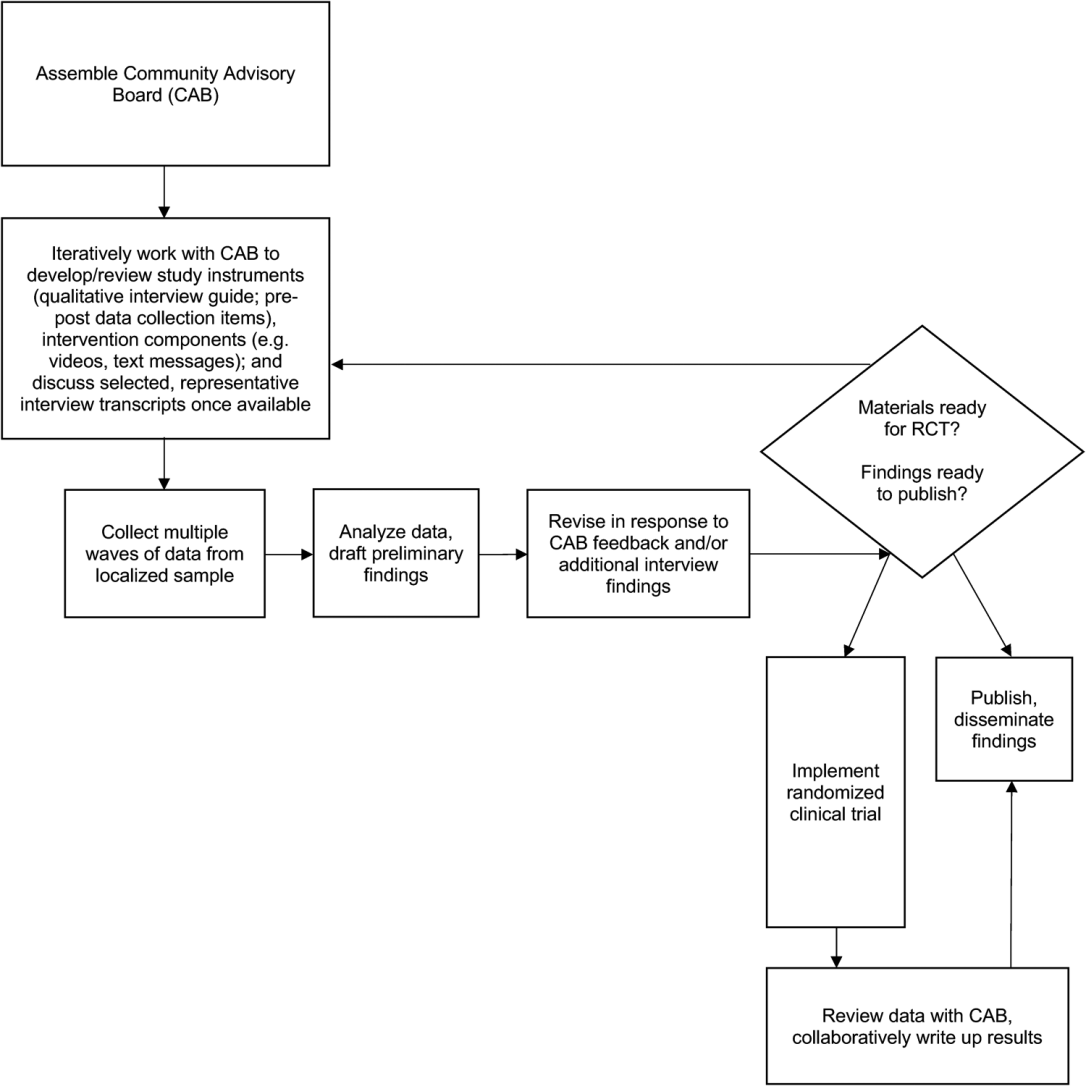
Process overview figure.

After each wave of interviews, we met with the CAB to discuss the data we had collected thus far, asking members for their responses to sections of interview transcripts and our accompanying analyses. We then incorporated CAB feedback into our emerging manuscripts as we wrote up our findings, and also relied on input from the CAB to shape new questions for upcoming waves of interviews. This process proved especially helpful in the development of intervention video content, and the subsequent evaluation (described further in the next section). Likewise, while we were writing the current paper, we presented draft descriptions of CAB comments to the group to be sure we accurately captured their perspectives on our work. Please see Figure 1 for a detailed illustration.

### Development and evaluation of the video intervention

To ensure participants understood key facts about COVID-19 and related vaccinations, we decided to work with two highly accomplished peer educators who had appeared in videos we created for previous studies (2, 12). For the current project we recorded both standing in recognizable outdoor settings around the neighborhood served by our community-based partner organization. Both spoke into the camera, as if they were speaking directly to the viewer, and provided details to address gaps in health literacy identified during our first two rounds of interviews (e.g., the vaccine does not contain the COVID-19 virus, so it is not possible to contract COVID-19 from a vaccination; COVID-19 is far more deadly compared to influenza, and has killed well over 10 times as many people each year in the United States). Because our peer educators are both native Spanish speakers, we recorded them delivering each point of information in English and then again in Spanish.

To address more specific clinical concerns (i.e., who should vaccinate; whether people with compromised immune systems were better off avoiding the vaccine) we recorded a nurse who has worked with our community partner organization for many years. She appears onscreen in a clinical examination room speaking to someone who pretends to be a patient (we did not use an actual patient to avoid privacy concerns). Because the nurse does not speak Spanish, we recorded her speaking in English and then worked with an actor to record her dialog as Spanish voice over.

As noted earlier, participant interviews repeatedly mentioned that featuring community members who appeared onscreen describing why they vaccinated would encourage viewers to vaccinate, especially if the onscreen community members emphasized that they didn't suffer side effects. Therefore, we decided to seek out community members who could address key facilitators that emerged during earlier phases of our research. These included: explaining they vaccinated to protect family members; offering reassurance that the vaccine was not something to fear; and explicitly saying they did not experience any severe post-vaccination side effects.

At a CAB meeting just before we started recording our peer educators and nurse, we asked CAB members if they could recommend any community members who might be willing to appear in our video and speak to the themes that emerged above. We envisioned that CAB members would refer us to individuals with compelling stories. Instead, all the CAB members present at the meeting volunteered to appear in the video. Given that they all originally declined vaccination, and some remained firmly opposed to vaccination for significant amounts of time, they made exceptionally strong contributions to our intervention video.

### Summative evaluation, 3rd wave of qualitative research

Qualitative data show the third wave of interview participants liked the video intervention we showed them, and in particular, appreciated the sequences addressing health literacy issues identified during initial rounds of our qualitative research. Interview participants had especially strong, positive reactions to a peer educator who looks into the camera and tells viewers that the vaccine does not contain a sample of the virus, and explains that, therefore, “you can't get COVID-19 just by receiving the vaccine.” Similar to participants who evaluated our storyboards, participants who watched the video reported that learning this encouraged them to re-consider vaccination.

“That's what I thought; um, and it's good to know that isn't the case because … I thought it was the other way around and that's why I didn't- that's what made me think, okay, I probably shouldn't do that because you don't know if the virus can come back to life or something or if it's not completely dead.”—Interviewee 48, White Hispanic Male.

Additionally, participants reported being engaged by another sequence in which a different peer educator appears onscreen and explains that more than 800,000 people in the United States had already been killed by COVID-19 since 2020, in contrast to influenza, which kills about 30,000 people each year nationwide. Our findings show people not only re-evaluated their decisions not to vaccinate after watching this part of the video, but interview data suggest this content encouraged participants to watch the rest of the video more carefully, becoming increasingly open to our intervention messaging.

The one that was actually talking the numbers. You know, cause that, that, that, that shows a lot … The one … that said that, ‘All right. We had 800,000’ … 800,000 deaths. I thought he was moving. You know, he was perfect because he was throwing numbers out there, you know, and trying to put facts out there, you know? Whether they're facts or whatever the case may be. Um, I felt, you know, his, the way he described it, it was, it was, you know, it was cool on his behalf.—Interviewee 50, Black Hispanic Male.

**Figure 2:**
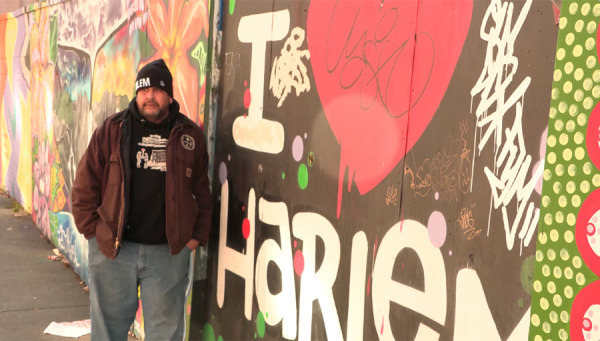
Screen capture from our intervention video depicting a peer educator who discusses the importance of COVID vaccination. All people who appear in the video, and the settings in which they appear, were selected after extensive input from participants and our community advisory board. Likewise, the messages people deliver onscreen were also developed with participant and CAB input.

Similar to the interview participants, members of our CAB also responded enthusiastically to the video overall, and especially to the representation of people they recognized as community members—this included people they already knew, as well as those they didn't (Figure 2 shows a screen capture from our video in which a well-known peer educator describes his own experience to underscore the importance of vaccination).

One CAB member praised the outdoor locations included in the video, which showed recognizable locations in Harlem. He told us that when he watches videos online, he looks for urban settings with apartment buildings in the video background that appear similar to the building he lives in, and that this is far more important than the skin color of anyone onscreen. Another CAB member who appears in the video intervention discussed the importance of clothing, explaining that he wanted to see a person wearing a “hoodie” and especially did not want to see a doctor wearing a “smock”.

Despite the overall positive response to our materials, at each step in our process some interview participants questioned the actual existence and severity of COVID. This theme is expressed particularly well in the following quote from a participant in our final round of interviews.

I was still going outside, even though, you know, we had to do quarantine … but I haven't caught COVID … nobody in my household has caught COVID neither, thank God … Sometimes I don't believe in it. … I hear about it, you know, that there's people that told me that they had loved ones that died from it or whatever the case may be and I'll be like, “Show me. Show me some proof. Let me know it's for real.”—Interviewee 50, Black Hispanic Male.

Additionally, data from each round of interviews show that some participants wondered if their drug use somehow might have protected them, or the people around them, from COVID, and if so, whether this further validated their decision not to vaccinate.

I know that people use drugs in the street. I see they don't get sick. It's weird, you know, it's weird, but I see a lot of people don't get sick. … I never see somebody die … that live in the street, with that. And they got contact with everything.—Interviewee 49, Hispanic Male.

Lastly, one participant in our final round of interviews speculated that vaccinating might even lessen her protection against COVID-19:

I've never got COVID and I, I, I've here in the streets for a whole three years and I've never got it, I mean, unless I didn't know it. So I don't know, I mean, I got a flu shot once and I got sick, I got really sick, so I'm afraid to get vaccinated.—Interviewee 43, White Non-Hispanic Female.

Our video intervention content is designed specifically to address these points. We developed two sections of the video in collaboration with the nurse who appears onscreen. We asked her why patients told her they had avoided vaccination and how we could address their concerns. The resulting content relates directly to the themes presented above. In one sequence, a nurse appears onscreen telling a patient that their immune system may be compromised due to drug use or “hard living”, and therefore, they need to vaccinate because they are most at risk from COVID. In a separate sequence, the nurse tells the patient that if they have not already gotten sick from COVID they are very lucky, but luck is finite and they now need a vaccination to protect their health.

### Quantitative evaluation

The next step in our research plan is to examine the effectiveness of our intervention materials *via* a clinical trial at the community-based syringe service program where we recruited interviewees, discussed above. Outcomes will include how many non-vaccinated participants agree to vaccinate after watching our intervention video, along with how many return for a required second dose and a recommended booster shot. This trial will enable thorough quantitative evaluations of our interventions to better understand how well our materials work overall, and whether some participants may respond better than others. For example, in a previous PEARLE-guided project we found that among emergency department patients aged 13–24, those who reported increased substance use or sexual risk were significantly more likely to accept an HIV test post-intervention (1) compared to participants who did not report increased substance use or sexual risk. We now plan to examine, among other comparisons, whether differences in participant vaccination rates emerge by self-reported demographic characteristics (e.g., age, race, primary spoken language) or differences in behavior (i.e., types of substances used, overdose risk). These quantitative findings can help inform future research and practice, in addition to helping us further refine the PEARLE methodology.

## Discussion

For years, interventionists have examined whether efforts to change health behavior are more effective if they are led by “experts”, such as physicians, or by members of a given community [for additional detail, please see Durantiini et al. (19)]. PEARLE, in contrast, aims to draw upon the expertise of community members to guide the design, development, and revision of technology-based interventions. Our inclusive, iterative process recognizes there are limits to the accumulated knowledge of professional researchers and intervention developers. Working in partnership with people who are members of, and possibly provide services to, a specific community enables us to better establish what content points we need to address (i.e., why people who inject drugs decline vaccination) and who can most credibly deliver our onscreen messages (e.g., a recognizable community member instead of a doctor in a smock). PEARLE also recognizes that each separate audience of target intervention recipients may have very different ideas about the credibility and applicability of intervention content, and therefore encourages new rounds of formative research not only when a new intervention is created, but each time an intervention is adapted for a new population or setting.

Additionally, the emphasis on iterative evaluation and revision in response to ongoing feedback from a sample of intended end-users enables us to identify specific areas we need to address in an intervention (e.g., fears of dangerous vaccination side effects), how we should best address each one (showing community members on camera who discuss their vaccination experience and emphasize they did not get sick), and what we should avoid (images of syringes that may invoke negative emotional responses).

Perhaps most importantly, just as PEARLE helps us as intervention developers get to know a community, our methodology helps community stakeholders get to know us. Each interview, CAB meeting, and casual interaction provides opportunities to show respect to participants, learn from them, and collaboratively shape our materials in response to themes that emerge from our discussions. By doing so, we seek to earn the trust of the populations we are trying to reach. This becomes especially salient during current discussions of equity in research and the dynamics between community members and “outside researchers”. Although there are multiple frameworks for implementation research and examinations of existing products [e.g., CFIR (20)] and well established models for conducting community engaged research [i.e., CBPR (21)] to the best of our knowledge, there is no comparable set of guidelines for the design and development of technology-based intervention content. As noted above, the main purpose of the current paper is to document each step of the process in which our team used the PEARLE methodology to create intervention content in English and Spanish. We now hope our methods can be adopted by other intervention developers addressing additional behavioral health issues with high-need populations nationwide and internationally.

## Data Availability

The original contributions presented in the study are included in the article, further inquiries can be directed to the corresponding author.
